# Scattered manifold-valued data approximation

**DOI:** 10.1007/s00211-016-0823-0

**Published:** 2016-07-08

**Authors:** Philipp Grohs, Markus Sprecher, Thomas Yu

**Affiliations:** 1 Fakultät für Mathematik, Universität Wien, Oskar-Morgenstern Platz 1, 1090 Wien, Austria; 2 Seminar for Applied Mathematics, ETH Zürich, Rämistrasse 101, 8092 Zürich, Switzerland; 3 Department of Mathematics, Drexel University, 3141 Chestnut Street, Korman 269, Philadelphia, PA 19104 USA

**Keywords:** Riemannian data, Manifold-valued function, Approximation, Scattered data, Model reduction, Primary 65D07, Secondary 65D15, 53B20, 41AXX, 35RXX

## Abstract

We consider the problem of approximating a function *f* from an Euclidean domain to a manifold *M* by scattered samples $(f(\xi _i))_{i\in \mathcal {I}}$, where the data sites $(\xi _i)_{i\in \mathcal {I}}$ are assumed to be locally close but can otherwise be far apart points scattered throughout the domain. We introduce a natural approximant based on combining the moving least square method and the Karcher mean. We prove that the proposed approximant inherits the accuracy order and the smoothness from its linear counterpart. The analysis also tells us that the use of Karcher’s mean (dependent on a Riemannian metric and the associated exponential map) is inessential and one can replace it by a more general notion of ‘center of mass’ based on a general retraction on the manifold. Consequently, we can substitute the Karcher mean by a more computationally efficient mean. We illustrate our work with numerical results which confirm our theoretical findings.

## Introduction

Let $f :\Omega \subset \mathbb {R}^s \rightarrow M$ with *M* a Riemannian manifold be an unknown function and we only know its values at a set of distinct points $(\xi _i)_{i\in \mathcal {I}}\subset \bar{\Omega }$. We are concerned with finding an approximant to *f*. Such an approximation problem for manifold-valued data arises in numerical geometric integration [23], diffusion tensor interpolation [7] and more recently in fast online methods for dimensionality reduced-order models [5]. In a Stanford press release associated with the aeronautical engineering publications [3–5], it is emphasized that being able to accurately and efficiently interpolate on manifolds is a key to fast online prediction of aerodynamic flutter, which, in turn, may help saving lives of pilots and passengers.

In the aforementioned references it was implicitly assumed that the data points $(f(\xi _i))_{i\in \mathcal {I}} \in M$ are close enough so that they can all be mapped to a single tangent space $T_pM$ by the inverse exponential map $\log$. In these previous works the base point $p \in M$ is typically one of $(f(\xi _i))_{i\in \mathcal {I}}$ and the choice can be quite arbitrary. In this setting, the problem simply reduces to a linear approximation problem on the tangent space $T_pM$. To approximate the value $f(x) \in M$, use any standard linear method (polynomial, spline, radial basis function etc.) to interpolate the values $(\log (p,(f(\xi _i)))_{i\in \mathcal {I}}\subset T_pM$ at the abscissa $(\xi _i)_{i\in \mathcal {I}}$, then evaluate the interpolant *Q* at the desired value *x*, and finally apply the exponential map to get the approximation $f(x) \sim \exp (p,Q(x))$.

This ‘push-interpolate-pull’ technique only works when all the available data points $(f(\xi _i))_{i\in \mathcal {I}}$ fall within the injectivity radius of the point *p*, and the method only provides an approximation for *f*(*x*) if *x* is near *p*. In this case the problem is local, and the topology of the manifold plays no role. One may then question what would be the difference if one uses the push-interpolate-pull approach but with the exponential map replaced by an arbitrary chart. With the exponential map, the push-interpolate-pull method respects the symmetry, if any, of the manifold. However, it is not clear what is the practical advantage of the latter and if respecting symmetry is not the main concern, one is free to replace the exponential map by a retraction (see, e.g., [1, 11]) on the manifold.

The problem is more challenging when we have available data points $(f(\xi _i))_{i\in \mathcal {I}} \in M$ scattered at different parts of the manifold, and the manifold has a nontrivial topology. In this setting, we desire an approximation method with the following properties:

(i) The method is well-defined as long as the scattered data sites are reasonably close locally, but can otherwise be far apart globally.
(ii) The approximant should provide a decent approximation order when *f* is sufficiently smooth. Since standard approximation theory (see for example chapter 3 of [9]) tells us that for linear data there are methods which provide an accuracy of $O(h^m)$ when *f* is $C^m$ smooth, and $h=\min _{i\ne j} |\xi _i-\xi _j|$ is the global meshwidth, it is natural to ask for a method for manifold-valued data with the same accuracy order.
(iii) The approximant itself should be continuous.
(iv) The approximant should be efficiently computable for any given *x*.

Such a method ought to ‘act locally but think globally’, meaning that the approximating value should only depend on the data $f(\xi _i)$ for $\xi _i$ near *x*, but yet the approximant should be continuous and accurate for all *x* in the domain. This forces the method to be genuinely nonlinear.

In the shift-invariant setting, i.e. when $\Omega = \mathbb {R}^s$ and $(\xi _i)_{i\in \mathcal {I}}=h\mathbb {Z}^s$, the above problem was solved successfully first by a subdivision technique [13, 32] and more recently by a method based on combining a general quasi-interpolant with the Karcher mean [14]. Even more recently, the work [17] used a projection-based approach to generalize B-spline quasi-interpolation for regularly spaced data. In either case, it was shown that an approximation method for linear data can be suitably adapted to manifold-valued data without jeopardizing the accuracy or smoothness properties of the original linear method. This kind of (smoothness or approximation order) equivalence properties are analyzed by a method known as proximity analysis. It is also shown that in certain setups, the smoothness or approximation order equivalence can breakdown in unexpected ways [10, 11, 17, 30–32].

Note that all the previous work assumes ‘structured data’ in the sense that samples are taken on a regular grid or the nodes of a triangulation of a domain. However, there exist several applications (for instance in model reduction applications [3–5]) where such structured samples are not available but only scattered samples are provided.

The case of scattered data interpolation has not been treated so far and in this paper we provide a solution to the above problem in the multivariate scattered data setting. More precisely we combine the ideas of [14, 16] with the classical linear theory of scattered data approximation [29] to arrive at approximants which satisfy (i)-(iv) above.

We give a brief outline of this work. The following Sect. 2 presents a brief overview of approximation results for scattered data approximation of Euclidean data. Then in Sect. 3 we present our generalization of the linear theory to the manifold-valued setting. This section also contains our main result regarding the approximation power of our nonlinear construction. It turns out that our scheme retains the optimal approximation rate as expected from the linear case. This is formalized in Theorem 3.5. In this theorem the dependence of the approximation rate on the geometry of *M* is made explicit and appears in form of norms of iterated covariant derivatives of the $\log$ function of *M*. To measure the smoothness of an *M*-valued function we utilize a smoothness descriptor introduced in [16] and which forms a natural generalization of Hölder norms to the manifold-valued setting. Our results also hold true for arbitrary choices of retractions. We discuss this extension in Sect. 3.3. Finally in Sect. 4 we present numerical experiments for the approximation of functions with values in the sphere and in the manifold of symmetric positive definite (SPD) matrices. In all cases the approximation results derived in Sect. 3 are confirmed. We also examine an application to the interpolation of reduced order models and compare our method to the method introduced in [6] where it turns our that our method delivers superior approximation power.

## Scattered data approximation in linear spaces

In this section we present classical results concerning the approximation of scattered data in Euclidean space. Our exposition mainly follows the monograph [29].

### General setup

We start by describing a general setup for scattered data approximation. Let $\Omega \subset$ be an open domain and $\bar{\Omega }$ its closure. Let $\Xi = (\xi _i)_{i\in \mathcal {I}}\subset \bar{\Omega }\subset \mathbb {R}^s$ be a set of sites and $\Phi :=(\varphi _i)_{i\in \mathcal {I}}\subset C_c(\bar{\Omega },\mathbb {R})$ a set of basis functions, where $C_c(\bar{\Omega },\mathbb {R})$ denotes the set of compactly supported real-valued continuous functions on $\bar{\Omega }$. The linear quasi-interpolation procedure, applied to a continuous function $f:\bar{\Omega }\rightarrow \mathbb {R}$, is defined as

1 $$\begin{aligned} \mathbf {Q} f(x):=\sum _{i\in \mathcal {I}} \varphi _i(x)f(\xi _i) \quad \text { for all } x\in \bar{\Omega }. \end{aligned}$$

Essential for the approximation power of the operator $\mathbf {Q}$ is the property that polynomials up to a certain degree are reproduced:

#### Definition 2.1

The pair $(\Xi ,\Phi )$ reproduces polynomials of degree *k* if

2 $$\begin{aligned} \sum _{i\in \mathcal {I}}\varphi _i(x)p(\xi _i) =p(x)\quad \text{ for } \text{ all } p\in \mathcal {P}_k(\mathbb {R}^s) \text { and }x \in \bar{\Omega }, \end{aligned}$$

where $\mathcal {P}_k(\mathbb {R}^s)$ denotes the space of polynomials of (total) degree $\le k$ on $\mathbb {R}^s$.

If $(\Xi ,\Phi )$ reproduces polynomials of degree $k\ge 0$ then necessarily

3 $$\begin{aligned} \sum _{i\in \mathcal {I}}\varphi _i(x) = 1\quad \text{ for } \text{ all } x\in \bar{\Omega }. \end{aligned}$$

#### Definition 2.2

We define the local meshwidth $h:\bar{\Omega } \rightarrow \mathbb {R}_{\ge 0}$ by

$$\begin{aligned} h(x) := \sup _{i\in \mathcal {I}(x)} |\xi _i - x|, \end{aligned}$$

where

$$\begin{aligned} \mathcal {I}(x) := \{i \in \mathcal {I}|\varphi _i(x)\ne 0\} \end{aligned}$$

and $|\cdot |$ denotes the Euclidean norm.

The following example gives a simple procedure to interpolate univariate data with polynomial reproduction degree 1.

#### Example 2.3

Let $$\Omega =(0,1)$$, $$0=\xi _1<\xi _2\dots <\xi _n=1$$ and

$$\begin{aligned} \varphi _i(x):=\left\{ \begin{array}{ll} \frac{x-\xi _{i-1}}{\xi _{i}-\xi _{i-1}} &{}\quad \text {if }i>1 \text { and } \xi _{i-1}\le x<\xi _i\\ \frac{\xi _{i+1}-x}{\xi _{i+1}-\xi _{i}} &{}\quad \text {if }i<n \text { and } \xi _{i}\le x<\xi _{i+1}\\ 0 &{} \quad \text {otherwise} \end{array}.\right. \end{aligned}$$

These functions, also known as hat functions, reproduce polynomials up to degree 1. The local meshwidth for $\xi _i\le x < \xi _{i+1}$ is

$$\begin{aligned} h(x)=\left\{ \begin{array}{ll} 0 &{}\quad x=\xi _i\\ \xi _{i+1}-x &{}\quad \xi _i<x\le \frac{\xi _i+\xi _{i+1}}{2} \\ x-\xi _i &{} \quad \frac{\xi _i+\xi _{i+1}}{2}<x<\xi _{i+1} \end{array}.\right. \end{aligned}$$

We define the $C^k$ seminorm of $f:\Omega \rightarrow \mathbb {R}$ on $U\subset \Omega$ by

$$\begin{aligned} |f|_{C^k(U,\mathbb {R})}:=\sup _{x\in U} \sup _{\begin{array}{c} \mathbf {l}\in \mathbb {N}^s\\ |\mathbf {l}|=k \end{array} } |\partial ^{\mathbf {l}} f(x)|, \end{aligned}$$

where for $\mathbf {l}=(l_1,\dots ,l_s)\in \mathbb {N}^s$ we define $|\mathbf {l}|:=\sum _{i=1}^s l_i$ and

$$\begin{aligned} \partial ^{\mathbf {l}} f(x):=\frac{\partial ^{|\mathbf {l}\;|}f(x)}{\prod _{i=1}^s \left( \partial x_i\right) ^{l_i}}. \end{aligned}$$

Additionally we define $\partial ^{\mathbf {l}}f=f$ if $\mathbf {l}=(0,\dots ,0)$. To estimate the approximation error at $x\in \Omega$ we use the set

$$\begin{aligned} \Omega _x:=\bigcup _{i\in \mathcal {I}(x)} \left\{ (1-t)x+t\xi _i\ |\ t\in [0,1) \right\} . \end{aligned}$$

Note that if $\Omega$ is convex we have $\Omega _x\subset \Omega$ for all $x\in \Omega$. The following result gives a bound for the approximation error at $x\in \Omega$ in terms of the local meshwidth, the polynomial reproduction degree and the $C^k$ seminorm of *f* on $\Omega _x$.

#### Theorem 2.4

Let $\Omega \subset \mathbb {R}^s$ be an open domain, $k>0$ a positive integer, $\Xi = (\xi _i)_{i\in \mathcal {I}}\subset \bar{\Omega }\subset \mathbb {R}^s$ a set of sites and $\Phi =(\varphi _i)_{i\in \mathcal {I}}\subset C_c(\bar{\Omega },\mathbb {R})$ a set of basis functions. Assume that $(\Xi ,\Phi )$ reproduces polynomials of degree smaller than *k*. Then there exists a constant $C>0$, depending only on *k* and *s* such that for all $f\in C^k(\Omega ,\mathbb {R})$ and $x\in \Omega$ with $\Omega _x\subset \Omega$ and $|f|_{C^k(\Omega _x,\mathbb {R})}<\infty$ we have

4 $$\begin{aligned} |f(x) - \mathbf {Q}f(x)| \le C \sum _{i\in \mathcal {I}}|\varphi _i(x)|\ |f|_{C^k(\Omega _x,\mathbb {R})}h(x)^k. \end{aligned}$$

We omit a proof here as a general theorem will be proven in the next section. If $\Omega _x \varsubsetneq \Omega$ we can consider an extension operator $E_x:C^k(\Omega ,M)\rightarrow C^k(\Omega \cup \Omega _x,M)$. Then the estimate (4) with a constant *C* also depending on the domain $\Omega$ holds true (see Remark 3.6). In contrast to other results, such as those in [29], Theorem 2.4 poses no restrictions on the sites $\Xi = (\xi _i)_{i\in \mathcal {I}}$. In the following subsection we will show how the approximation results in [29] follow as a corollary to the previous Theorem 2.4.

### Moving least squares

In this subsection, we will follow [29] and show how to construct a set of compactly supported basis functions $\Phi =(\varphi _i)_{i \in \mathcal {I}}$ with polynomial reproduction degree *k* for a set of sites $\Xi = (\xi _i)_{i\in \mathcal {I}}\subset \bar{\Omega }\subset \mathbb {R}^s$ which is $(k,\delta )$-unisolvent.

#### Definition 2.5

A set of sites $\Xi = (\xi _i)_{i\in \mathcal {I}}\subset \bar{\Omega }\subset \mathbb {R}^s$ is called $(k,\delta )$-unisolvent if there exists no $x\in \bar{\Omega }$ and $p\in \mathcal {P}_k(\mathbb {R}^s)$ with $p\ne 0$ and $p(\xi _i)=0$ for all $i \in \mathcal {I}$ with $|\xi _i-x|\le \delta$.

Let $\alpha :[0,\infty ) \rightarrow [0,1]$ with $\alpha (0)=1,\ \alpha (z)=0$ for $z\ge 1$ and $\alpha '(0)=\alpha '(1)=0$, e.g. the Wendland function defined by

$$\begin{aligned} \alpha (x):=\left\{ \begin{array}{ll} (1+4x)(1-x)^4 &{}\quad 0\le x \le 1\\ 0 &{}\quad x>1 \end{array}.\right. \end{aligned}$$

For every $i\in \mathcal {I}$ and $x\in \bar{\Omega }$ we define

5 $$\begin{aligned} \varphi _i(x):=\alpha \left( \frac{|x-\xi _i|}{\delta }\right) p(\xi _i) \end{aligned}$$

where $p \in \mathcal {P}_k(\mathbb {R}^s)$ is chosen such that the basis functions $\Phi =(\varphi _i)_{i \in \mathcal {I}}$ satisfy the polynomial reproduction property (2) of degree *k*, i.e.

6 $$\begin{aligned} \sum _{j \in \mathcal {I}} \alpha \left( \frac{|x-\xi _j|}{\delta }\right) p(\xi _j)q(\xi _j)=q(x) \qquad \forall \; q \in \mathcal {P}_k(\mathbb {R}^s). \end{aligned}$$

As $\Xi$ is $(k,\delta )$-unisolvent the left hand side of (6) can be regarded as an inner product of *p* and *q*. Furthermore $q\rightarrow q(x)$ is a linear functional on $\mathcal {P}_k(\mathbb {R}^s)$. Hence by the Riesz representation theorem there exists a unique polynomial $p\in \mathcal {P}_k(\mathbb {R}^s)$ with (6).

Note that choosing a basis on $\mathcal {P}_k(\mathbb {R}^s)$ one can compute *p* by solving a linear system of equations. Hence we can compute $\varphi (x)$ in a reasonable time and $\mathbf {Q}$ satisfies (iv) from the introduction. In [29] it is shown that the function $\varphi$ has the same smoothness as $\alpha$. This shows that $\mathbf {Q}$ satisfies (iii) from the introduction.

The theory in [29] considers a quasi-uniform set of sites $\Xi$ as defined below.

#### Definition 2.6

The pair $(\Xi ,\delta )$ is called quasi-uniform with constant $c>0$ if

$$\begin{aligned} \delta \le c q_{\Xi }, \end{aligned}$$

where $q_{\Xi }$ is the separation distance defined by

$$\begin{aligned} q_{\Xi }:= & {} \frac{1}{2} \min _{\begin{array}{c} i,j \in \mathcal {I}\\ i \ne j \end{array}} |\xi _i-\xi _j|. \end{aligned}$$

For a shorter notation we introduce the symbol $[n] := \{1,\dots , n\}$.

#### Example 2.7

Let $\Omega =(-1,1)$ and $k, n\in \mathbb {N}$. For $i \in [n]$ we choose the node $\xi _i$ uniformly at random in the interval $(-1+2(i-1)/n,-1+2i/n)$ and $\delta =2(k+1)/n$. Then the set of sites $\Xi =(\xi _i)_{i \in [n]}$ is $(k,\delta )$-unisolvent. However, in order to make $\Xi$ quasi-uniform the constant $c>0$ can get arbitrarily large as $q_\Xi$ can get arbitrarily small.

#### Example 2.8

Let $\Omega =(-1,1)$ and $k,n\in \mathbb {N}$. For $i \in [n]$ we choose the node $\xi _i$ uniformly at random in the interval $[-1+(4i-3)/(2n),-1+(4i-1)/(2n)]$ and $\delta =(2k+3)/n$. Then the set of sites $\Xi =(\xi _i)_{i\in [n]}$ is $(k,\delta )$-unisolvent and quasi-uniform with $c=2(2k+3)$.

The following theorem is the main result of [29] regarding the approximation with moving least squares.

#### Theorem 2.9

[29] Let $\Omega \subset \mathbb {R}^s$ be an open and convex domain, $k>0$ a positive integer, $\Xi = (\xi _i)_{i\in \mathcal {I}}\subset \bar{\Omega }\subset \mathbb {R}^s$ a $(k-1,\delta )$-unisolvent set of sites and $\Phi =(\varphi _i)_{i\in \mathcal {I}}\subset C_c(\bar{\Omega },\mathbb {R})$ the basis functions as defined in (5). Let $\delta >0$ and assume that $(\Xi ,\delta )$ is quasi-uniform with $c>0$. Then there exists a constant $C>0$, depending only on *c*, *k* and *s* such that for all $f\in C^k(\Omega ,\mathbb {R})$ with $|f|_{C^k(\Omega ,\mathbb {R})}<\infty$ we have

$$\begin{aligned} \Vert f - \mathbf {Q}f\Vert _{L^{\infty }(\Omega )} \le C |f|_{C^k(\Omega ,\mathbb {R})}\delta ^k. \end{aligned}$$

We show that Theorem 2.9 is a consequence of Theorem 2.4.

#### Proof

Note that $h(x)=\sup _{i\in \mathcal {I}(x)} |\xi _i - x| \le \delta$. Due to [29, Theorem 4.7(2)] $\sup _{i\in \mathcal {I}(x)}|\varphi _i(x)|$ can be bounded by a constant depending only on *k*, *c* and *s*. We now show that $|\mathcal {I}(x)|$ can be bounded by a constant depending only on *c* and *s*. Note that

$$\begin{aligned} \mathcal {I}(x)\subseteq \{i \in \mathcal {I}\ |\ |x-\xi _i|\le \delta \}. \end{aligned}$$

Hence the pairwise disjoint balls with centers $(\xi _i)_{i \in \mathcal {I}(x)}$ and radii $q_{\Xi }$ lie in the ball with center *x* and radius $\delta +q_{\Xi }$. As the fraction $(\delta +q_{\Xi })/q_{\Xi }$ is bounded from above by $(1+c)$ we have that $\sup _{x \in \Omega }\ |\ \mathcal {I}(x)|$ is bounded by the number of balls with radius 1 that fit into a ball of radius $(1+c)$. In the following we use the letter *C* as a symbol for a constant whose value may change from equation to equation. By Theorem 2.4 we have,

$$\begin{aligned} \Vert f - \mathbf {Q}f\Vert _{L^{\infty }(\Omega )}= & {} \sup _{x \in \Omega } |f(x) - \mathbf {Q}f(x)|\\\le & {} \sup _{x \in \Omega } C \sum _{i\in \mathcal {I}(x)}|\varphi _i(x)| |f|_{C^k(\Omega _x,\mathbb {R})}h(x)^k\\\le & {} \sup _{x \in \Omega } C |\mathcal {I}(x)| \sup _{i\in \mathcal {I}}|\varphi _i(x)||f|_{C^k(\Omega ,\mathbb {R})}\delta ^k\\\le & {} C |f|_{C^k(\Omega ,\mathbb {R})}\delta ^k. \end{aligned}$$

$$\square $$

## Scattered data approximation in manifolds

The present section extends Theorems 2.4 and 2.9 to the case of manifold-valued functions $f:\Omega \rightarrow M$ with a Riemannian manifold *M*. First, in Sect. 3.1 we present a geometric generalization of the operator $\mathbf {Q}$ to the manifold-valued case. The construction is based on the idea to replace affine averages by a Riemannian mean as studied e.g. in [21]. Subsequently, in Sect. 3.2 we study the resulting approximation error. Finally, in Sect. 3.3 we extend our results to the case of more general notions of geometric average, induced by arbitrary retractions as studied e.g. in [15].

### Definition of Riemannian moving least squares

In this section we consider a Riemannian manifold *M* with distance metric $d :M\times M \rightarrow \mathbb {R}_{\ge 0}$ and metric tensor *g*, inducing a norm $|\cdot |_{g(p)}$ on the tangent space $T_pM$ at $p\in M$.

Extending the classical theory which we briefly described in Sect. 2 we now aim to construct approximation operators for functions $f:\Omega \rightarrow M$. We follow the ideas of [14, 16, 26–28] where the sum in (1) is interpreted as a weighted mean of the data points $(f(\xi _i))_{i\in \mathcal {I}(x)}$. Due to (3) this is justified.

For weights $\Gamma =(\gamma _i)_{i\in \mathcal {I}}\subset \mathbb {R}$ with $\sum _{i\in \mathcal {I}}\gamma _i = 1$ and data points $\mathcal {P}=(p_i)_{i\in \mathcal {I}}\subset M$ we can define the Riemannian average

7 $$\begin{aligned} av_M(\Gamma ,\mathcal {P}):= \mathop {\mathrm {argmin}}_{p\in M} \sum _{i\in \mathcal {I}}\gamma _i d(p,p_i)^2. \end{aligned}$$

One can show [21, 28] that $av_M(\Gamma ,\mathcal {P})$ is a well-defined operation, if the diameter of the set $\mathcal {P}$ is small enough:

#### Theorem 3.1

([2, 28]) Given a weight sequence $\Gamma =(\gamma _i)_{i\in \mathcal {I}}\subset \mathbb {R}$ with $\sum _{i\in \mathcal {I}}\gamma _i = 1$. Let $p_0\in M$ and denote for $\rho >0$ by $B_\rho$ the geodesic ball of radius $\rho$ around $p_0$. Then there exist $0<\rho _1\le \rho _2<\infty$, depending only on $\sum _{i\in \mathcal {I}}|\gamma _i|$ and the geometry of *M* such that for all points $\mathcal {P}=(p_i)_{i\in \mathcal {I}}\subset B_{\rho _1}$ the functional $\sum _{i\in \mathcal {I}}\gamma _i d(p,p_i)^2$ assumes a unique minimum in $B_{\rho _2}$.

Whenever the assumptions of Theorem 3.1 hold true, the Riemannian average is uniquely determined by the first order condition

8 $$\begin{aligned} \sum _{i\in \mathcal {I}} \gamma _i\log \left( av_M(\Gamma ,\mathcal {P}),p_i\right) = 0, \end{aligned}$$

which could alternatively be taken as a definition of the weighted average in *M*, see also [28].

We can now define an *M*-valued analogue for Eq. (1).

#### Definition 3.2

Denoting $\Phi (x):=(\varphi _i(x))_{i\in \mathcal {I}}\subset \mathbb {R}$ and $f(\Xi ):=(f(\xi _i))_{i\in \mathcal {I}}\subset M$ we define the nonlinear moving least squares approximant

$$\begin{aligned} \mathbf {Q}^Mf(x):= av_M(\Phi (x),\quad f(\Xi ))\in M. \end{aligned}$$

It is clear that in the linear case this definition coincides with (1). Furthermore it is easy to see that the smoothness of the basis functions $\Phi =(\varphi _i)_{i\in \mathcal {I}}$ gets inherited by the approximation procedure $\mathbf {Q}^M$, see e.g. [27]. Hence our approximation operator $\mathbf {Q}^M$ satisfies (iii) from the introduction.

#### Remark 3.3

We wish to emphasize that the approximation procedure as defined in Definition 3.2 is completely geometric in nature. In particular it is invariant under isometries of *M*. In mechanics this leads to the desirable property of objectivity. The push-interpolate-pull described in the introduction does in general not have this property.

### Approximation error

We now wish to assess the approximation error of the nonlinear operator $\mathbf {Q}^M$ and generalize Theorem 2.4 to the *M*-valued case.

#### The smoothness descriptor of manifold-valued functions

Two basic things need to be considered to that end. First we need to decide how we measure the error between the original function *f* and its approximation $\mathbf {Q}^Mf$. This will be done pointwise using the geodesic distance $d:M \times M \rightarrow \mathbb {R}_{\ge 0}$ on *M*. Slightly more subtle is the question what is the right analogue to the term $\Vert f\Vert _{C^k(\Omega ,\mathbb {R})}$ in the manifold-valued case? In [16] a so-called *smoothness descriptor* has been introduced to measure norms of derivatives of *M*-valued functions. Its definition requires the notion of covariant derivative in a Riemannian manifold. With $\frac{D}{dx^l}$ we denote the covariant partial derivative along *f* with respect to $x^l$. That is, given a function $f:\Omega \rightarrow M$ and a vector field $W:\Omega \rightarrow TM$ attached to *f*, i.e., $W(x)\in T_{f(x)}M$ for all $x\in \Omega$. Then in coordinates on *M*, the covariant derivative of *W* in $x^l$ reads

$$\begin{aligned} \frac{D}{dx^l}W^{r}(x):= \frac{dW^r}{dx^l}(x)+{\Gamma }_{ij}^{r}(f(x))\frac{df^{i}}{dx^l}W^{j}(x), \end{aligned}$$

where we sum over repeated indices and denote with ${\Gamma }_{ij}^{r}$ the Christoffel symbols associated to the metric of *M* [8]. For iterated covariant derivatives we introduce the symbol $\mathcal {D}^{{\mathbf {l}}}f$ which means covariant partial differentiation along *f* with respect to the multi-index $\mathbf {l}$ in the sense that

9 $$\begin{aligned} \mathcal {D}^{\mathbf {l}} f:=\frac{D}{dx^{l_k}} \dots \frac{D}{dx^{l_2}}\frac{df}{dx^{l_1}},\qquad \mathbf {l}\in [d]^k,\ k\in \mathbb {N}_0. \end{aligned}$$

Note that (9) differs from the usual multi-index notation, which cannot be used because covariant partial derivatives do not commute. The smoothness descriptor of an *M*-valued function is defined as follows.

##### Definition 3.4

For a function $f:\Omega \rightarrow M$, $k\ge 1$ and $U\subset \Omega$ we define the *k*-th order smoothness descriptor

$$\begin{aligned} \Theta _{k,U}(f):=\sum _{n=1}^k \sum _{\begin{array}{c} \mathbf {l}_j \in [d]^{m_j}\\ m_j>0, j\in [n]\\ \sum _{j=1}^n m_j = k \end{array} } \sup _{x\in U} \prod _{j=1}^n \bigg |\mathcal {D}^{{\mathbf {l}_j}}f(x)\bigg |_{g(f(x))}. \end{aligned}$$

The smoothness descriptor as defined above represents a geometric analogue of the classical notions of Hölder norms and seminorms. Note that, even in the Euclidean case (for instance $M=\mathbb {R}$) the expression $\Theta _{k,U}(f)$ is not equal to the Hölder seminorm $|f|_{C^k(U,\mathbb {R})}$, as additional terms are present in the definition of $\Theta _{k,U}(f)$. But we have the implications

$$\begin{aligned} \Theta _{k,U}(f)< \infty \Leftrightarrow |f|_{C^l(U,\mathbb {R})}< \infty \quad \text { for all } l\in [k]. \end{aligned}$$

In the proof of Theorem 3.5 it will become clear why the additional terms in $\Theta _{k,U}(f)$ are needed in the case of general *M* where, in contrast to $M=\mathbb {R}$, higher order covariant derivatives of the logarithm mapping need not vanish, compare also Remark 3.7 below.

#### Further geometric quantities

Coming back to the anticipated generalization of Theorem 2.4, we also aim to quantify exactly to which extent the approximation error depends on the geometry of *M*. To this end let $\log (p,\cdot ) :M \rightarrow T_pM$ be the inverse of the exponential map at *p*. Denote by $\nabla _1$, $\nabla _2$ the covariant derivative of a bivariate function with respect to the first and second argument, respectively. In particular, for $l\in \mathbb {N}$ we will require the derivatives

$$\begin{aligned} \nabla _2^l\log (p,q):(T_qM)^l \rightarrow T_pM \end{aligned}$$

and their norms

$$\begin{aligned} \Vert \nabla _2^l\log (p,q)\Vert =\sup _{v_1,\dots , v_l\in T_qM} \frac{\left| \nabla _2^l\log (p,q)\left( v_1,\dots , v_l\right) \right| _{g(p)}}{\prod _{i=1}^l|v_i|_{g(q)}}. \end{aligned}$$

#### Main approximation result

We now state and prove our main result.

##### Theorem 3.5

Let $\Omega \subset \mathbb {R}^s$ be an open domain, $k>0$ a positive integer, $\Xi = (\xi _i)_{i\in \mathcal {I}}\subset \bar{\Omega }\subset \mathbb {R}^s$ a set of sites and $\Phi =(\varphi _i)_{i\in \mathcal {I}}\subset C_c(\bar{\Omega },\mathbb {R})$. Assume that $(\Xi ,\Phi )$ reproduces polynomials of degree smaller than *k*. Then there exists a constant $C>0$, depending only on *k* and *s*, such that for all $f\in C^k(\Omega ,M)$ and $x\in \Omega$ with $\Omega _x\subset \Omega$ and $\Theta _{k,\Omega _x}(f)<\infty$ we have

10 $$\begin{aligned}&d\left( \mathbf {Q}^Mf(x),f(x)\right) \le C \Theta _{k,\Omega _x}(f) \sum _{i\in \mathcal {I}(x)}|\varphi _i(x)| \nonumber \\&\quad \times \sup _{1\le r\le k}\sup _{y\in \Omega _x} \left\| \nabla _2^{r}\log \left( \mathbf {Q}^Mf(x),f(y)\right) \right\| \left( h(x)\right) ^k. \end{aligned}$$

##### Remark 3.6

If $\Omega _x \varsubsetneq \Omega$ we can consider an extension operator (see e.g. [19]) $E_x:C^k(\Omega ,M)\rightarrow C^k(\Omega \cup \Omega _x,M)$. Then the estimate (10) with *f*(*y*) replaced by $E_xf(y)$ and a constant *C* also depending on the domain $\Omega$ holds true.

##### Proof

We shall use the balance law (8) which implies that we can write

11 $$\begin{aligned} \varepsilon (x)&:=\log \left( \mathbf {Q}^Mf(x),f(x)\right) \nonumber \\&= \log \left( \mathbf {Q}^Mf(x),f(x)\right) \nonumber \\&\quad -\sum _{i\in \mathcal {I}(x)}\varphi _i(x) \log \left( \mathbf {Q}^Mf(x),f(\xi _i)\right) . \end{aligned}$$

Now we consider the function $G:\Omega \times \Omega \rightarrow TM$ defined by

12 $$\begin{aligned} G(x,y):=\log \left( \mathbf {Q}^Mf(x),f(y)\right) \in T_{\mathbf {Q}^Mf(x)}M. \end{aligned}$$

Since, for fixed $x\in \Omega$, the function *G* maps into a linear space we can perform a Taylor expansion of *G* in *y* around the point $(x,x)\in \Omega \times \Omega$ and obtain

13 $$\begin{aligned} G(x,y) = \sum _{\begin{array}{c} \mathbf {l} \in \mathbb {N}^s\\ |\mathbf {l}\;| < k \end{array}}\frac{(y-x)^{\mathbf {l}}}{\mathbf {l}\;!} \partial _2^{\mathbf {l}}G(x,x) + \sum _{\begin{array}{c} \mathbf {l} \in \mathbb {N}^s\\ |\mathbf {l}\;|=k \end{array}}R_{\mathbf {l}}(x,y)(y-x)^{\mathbf {l}}, \end{aligned}$$

where for any $l=(l_1,\dots ,l_s) \in \mathbb {N}^s$ and $z=(z_1,\dots ,z_s) \in \mathbb {R}^s$ we define

$$\begin{aligned} z^{\mathbf {l}}:=\prod _{i=1}^s (z_i)^{l_i},\qquad \mathbf {l}\;!:=\prod _{i=1}^s l_i!,\qquad \partial _2^{\mathbf {l}}G(x,z):=\frac{\partial ^{|\mathbf {l}\;|}G(x,z)}{\prod _{i=1}^s \left( \partial z_i\right) ^{l_i}} \end{aligned}$$

and

14 $$\begin{aligned} R_{\mathbf {l}}(x,y):=\frac{|\mathbf {l}\;|}{\mathbf {l}\;!} \int _0^1 (1-t)^{|\mathbf {l}\;|-1}\partial _2^{\mathbf {l}}G (x,x+t(y-x))dt. \end{aligned}$$

We insert (13) into (11) and get the following expression for $\varepsilon (x)$:

15 $$\begin{aligned} \varepsilon (x) = G(x,x) - \sum _{i\in \mathcal {I}(x)}\varphi _i(x) \left( \sum _{\begin{array}{c} \mathbf {l} \in \mathbb {N}^s\\ |\mathbf {l}\;| < k \end{array}}\frac{(\xi _i-x)^{\mathbf {l}}}{\mathbf {l}\;!} \partial _2^{\mathbf {l}}G(x,x) + \sum _{\begin{array}{c} \mathbf {l} \in \mathbb {N}^s\\ |\mathbf {l}\;|=k \end{array}}R_{\mathbf {l}}(x,\xi _i)(\xi _i-x)^{\mathbf {l}} \right) \end{aligned}$$

Exchanging summation order in (15) yields

$$\begin{aligned} \varepsilon (x) = \underbrace{G(x,x) - \sum _{\begin{array}{c} \mathbf {l} \in \mathbb {N}^s\\ |\mathbf {l}\;| < k \end{array}} \sum _{i\in \mathcal {I}(x)}\varphi _i(x) \frac{(\xi _i-x)^{\mathbf {l}}}{\mathbf {l}\;!} \partial _2^{\mathbf {l}}G(x,x)}_{(I)} + \underbrace{ \sum _{\begin{array}{c} \mathbf {l} \in \mathbb {N}^s\\ |\mathbf {l}\;|=k \end{array}}\sum _{i\in \mathcal {I}(x)} \varphi _i(x)R_{\mathbf {l}}(x,\xi _i)(\xi _i-x)^{\mathbf {l}}}_{(II)}. \end{aligned}$$

We will show that $(I)=0$ and $(II)=O(h(x)^k)$ which implies our claim.

Let us start by showing that $(I)=0$. As a first observation we note that, due to (3), we can write

$$\begin{aligned} (I)=\sum _{\begin{array}{c} \mathbf {l} \in \mathbb {N}^s\\ 0<|\mathbf {l}\;| < k \end{array}} \underbrace{\sum _{i\in \mathcal {I}(x)}\varphi _i(x) \frac{(\xi _i-x)^{\mathbf {l}}}{\mathbf {l}\;!} \partial _2^{\mathbf {l}}G(x,x)}_{(I_{\mathbf {l}})}. \end{aligned}$$

We claim that $(I_{\mathbf {l}})=0$ for all $\mathbf {l}\in \mathbb {N}^s$ with $|\mathbf {l}\;|<k$. Indeed, pick $x_*\in \Omega$ arbitrary. Then, by the polynomial reproduction property (2) and $k\le m+1$ we get

16 $$\begin{aligned} \sum _{i\in \mathcal {I}(x)}\varphi _i(x) \frac{(\xi _i-x_*)^{\mathbf {l}}}{\mathbf {l}\;!} \partial _2^{\mathbf {l}}G(x_*,x_*) = \partial _2^{\mathbf {l}}G(x_*,x_*) \frac{(x-x_*)^{\mathbf {l}}}{\mathbf {l}\;!}\quad \text{ for } \text{ all } x,\,x_*\in \Omega . \end{aligned}$$

Setting $x_*= x$ in (16) yields

$$\begin{aligned} (I_{\mathbf {l}})=\sum _{i\in \mathcal {I}(x)}\varphi _i(x) \frac{(\xi _i-x)^{\mathbf {l}}}{\mathbf {l}\;!} \partial _2^{\mathbf {l}}G(x,x) = \partial _2^{\mathbf {l}}G(x,x) \frac{(x-x)^{\mathbf {l}}}{\mathbf {l}\;!} =0 \end{aligned}$$

which proves the desired claim.

We now move on to prove our second claim, namely that $(II)=O(h(x)^k)$. To this end we need to estimate, for any $\mathbf {l} \in \mathbb {N}^s$ with $|\mathbf {l}\;|=k$ the quantity

17 $$\begin{aligned} (II)_{\mathbf {l}}:=\sum _{i\in \mathcal {I}(x)} \varphi _i(x)R_{\mathbf {l}}(x,\xi _i)(\xi _i-x)^{\mathbf {l}}. \end{aligned}$$

To this end we consider, for fixed $\mathbf {l}$ and $i\in \mathcal {I}(x)$, the quantity $R_{\mathbf {l}}(x,\xi _i)$. In the following we use the letter *C* as a symbol for a constant whose value may change from equation to equation. Inserting Definition (12) and using the chain rule we obtain that

$$\begin{aligned} \left| \partial _2^{\mathbf {l}}G(x,y)\right| _{g\left( \mathbf {Q}^Mf(x)\right) }&\le C \sum _{\begin{array}{c} 1\le r\le k,\ \mathbf {l}\in [d]^{m}\\ \sum _{j=1}^{r}m = k \end{array}} \left| \nabla _2^{r}\log \left( \mathbf {Q}^Mf(x),f(y)\right) \right. \\&\left. \quad \times \,\Big (\mathcal {D}^{{\mathbf {l}}_1}f(y),\dots , \mathcal {D}^{{\mathbf {l}}_r}f(y)\Big )\right| _{g\left( \mathbf {Q}^Mf(x)\right) }, \end{aligned}$$

which can be estimated by

18 $$\begin{aligned} \left| \partial _2^{\mathbf {l}}G(x,y)\right| _{g\left( \mathbf {Q}^Mf(x)\right) }&\le C\sup _{1\le r\le k}\left\| \nabla _2^{r}\log \left( \mathbf {Q}^Mf(x),f(y)\right) \right\| \nonumber \\&\quad \times \sum _{\begin{array}{c} 1\le r\le k,\ \mathbf {l}_j\in [d]^{m_j} \nonumber \\ m_j>0, j\in [r] \nonumber \\ \sum _{j=1}^{r}m_j = k \end{array}} \left| \mathcal {D}^{{\mathbf {l}}_1}f(y)\right| _{g(f(y))}\cdot \dots \cdot \left| \mathcal {D}^{{\mathbf {l}}_r}f(y)\right| _{g(f(y))}\\&\le C\sup _{1\le r\le k}\left\| \nabla _2^{r}\log \left( \mathbf {Q}^Mf(x),f(y)\right) \right\| \Theta _{k,\Omega _x}(f). \end{aligned}$$

Inserting estimate (18) into the definition (14) of $R_{\mathbf {l}}$ we get that

19 $$\begin{aligned} \left| R_{\mathbf {l}}(x,\xi _i)\right| _{g\left( \mathbf {Q}^Mf(x)\right) }&\le C \Theta _{k,\Omega _x}(f) \int _0^1 (1-t)^{k-1} \nonumber \\&\quad \times \sup _{1\le r\le k}\left\| \nabla _2^{r} \log \left( \mathbf {Q}^Mf(x),f(x+t(\xi _i-x))\right) \right\| dt\nonumber \\&\le C \Theta _{k,\Omega _x}(f) \sup _{1\le r\le k}\sup _{y\in \Omega _x} \left\| \nabla _2^{r}\log \left( \mathbf {Q}^Mf(x),f(y)\right) \right\| . \end{aligned}$$

Finally, putting (19) into (17) we get that

20 $$\begin{aligned} \left| (II)_{\mathbf {l}}\right| _{g\left( \mathbf {Q}^Mf(x)\right) }&\le C \Theta _{k,\Omega _x}(f) \sup _{1\le r\le k}\sup _{y\in \Omega _x} \left\| \nabla _2^{r}\log \left( \mathbf {Q}^Mf(x),f(y)\right) \right\| \nonumber \\&\quad \times \sum _{i\in \mathcal {I}(x)} |\varphi _i(x)| |\xi _i - x|^{|\mathbf {l}\;|}\nonumber \\&\le C \Theta _{k,\Omega _x}(f) \sup _{1\le r\le k}\sup _{y\in \Omega _x} \left\| \nabla _2^{r}\log \left( \mathbf {Q}^Mf(x),f(y)\right) \right\| \nonumber \\&\quad \times \sum _{i\in \mathcal {I}(x)} |\varphi _i(x)|h(x)^k. \end{aligned}$$

Summing up over all $\mathbf {l}\in \mathbb {N}^s$ with $|\mathbf {l}\;| = k$ we get the desired estimate. $\square$

Two remarks are in order regarding Theorem 3.5.

##### Remark 3.7

Clearly, in the linear case higher order (i.e. higher than order 1) derivatives of the logarithm mapping $\log (p,q)=q-p$ vanish. Using this fact it is easy to see that our proof of Theorem 3.5 reduces to Theorem 2.4 in the linear case.

##### Remark 3.8

The estimate in Theorem 3.5 completely separates the error contributions of *f* and of the geometry of *M*. We thus see that the only geometric quantity which influences the approximation consists of iterated covariant derivatives of the logarithm mapping.

Using Theorem 3.5 we can now state and prove a geometric generalization to Wendland’s main theorem on moving least squares approximation, e.g., Theorem 2.9.

##### Theorem 3.9

Let $\Omega \subset \mathbb {R}^s$ be an open and convex domain, $k>0$ a positive integer, $\Xi = (\xi _i)_{i\in \mathcal {I}}\subset \bar{\Omega }\subset \mathbb {R}^s$ a $(k-1,\delta )$-unisolvent set of sites and $\Phi =(\varphi _i)_{i\in \mathcal {I}}\subset C_c(\bar{\Omega },\mathbb {R})$ the basis functions as defined in (5). Let $\delta >0$ and assume that $(\Xi ,\delta )$ is quasi-uniform with $c>0$. Then there exists a constant $C>0$, depending only on *c*, *k* and *s* such that for all $f\in C^k(\Omega ,M)$ with $\Theta _{k,\Omega }(f)<\infty$ we have

$$\begin{aligned} \sup _{x\in \Omega }d\left( f(x) ,\mathbf {Q}^Mf(x)\right) \le C \sup _{1\le r\le k}\sup _{x\in \Omega }\sup _{y\in \Omega _x} \left\| \nabla _2^{r}\log \left( \mathbf {Q}^Mf(x),f(y)\right) \right\| \Theta _{k,\Omega }(f)\delta ^k. \end{aligned}$$

##### Proof

The proof proceeds exactly as the proof of Theorem 2.9, using Theorem 3.5 instead of Theorem 2.4. $\square$

Our approximation operator $\mathbf {Q}^M$ satisfies (i) and (ii) from the introduction.

### Generalization to retraction pairs

The computation of the quasi-interpolant $\mathbf {Q}^Mf(x)$ requires the efficient computation of the exponential and logarithm mapping of *M*. For many practical examples of *M* this is not an issue, however in certain cases (for instance the Stiefel manifold [15]) it is computationally expensive to compute the exponential or logarithm function of a given manifold. Then, alternative functions can sometimes be used. This idea is formalized by the concept of *retraction pairs*.

#### Definition 3.10

([15]*, see also* [1, 12]) A pair (*P*, *Q*) of smooth functions

$$\begin{aligned} P:TM \rightarrow M,\quad Q:M\times M\rightarrow TM \end{aligned}$$

is called a *retraction pair* if

$$\begin{aligned} P\left( x,Q\left( x,y\right) \right) = y,\quad \text{ for } \text{ all } x,\ y\in M, \text{ and } \\ P\left( x,0\right) =x, \quad \frac{d}{dv} P(x,v)\Big |_{v=0} = \mathrm {Id} \quad \text{ for } \text{ all } x\in M. \end{aligned}$$

In general *P* may only be defined locally around *M*, and *Q* around the diagonal of $M\times M$.

#### Example 3.11

Certainly, the pair $(\exp ,\log )$ satisfies the above assumptions [8], and therefore forms a retraction pair. Let $S^m=\{x\in \mathbb {R}^{m+1}||x|=1\}$ be the *m*-dimensional sphere. Here we can define a retraction pair (*P*, *Q*) by

$$\begin{aligned} P(x,y)=\frac{x+y}{|x+y|} \quad \text { and }\quad Q(x,y)=\frac{y}{\langle x,y \rangle }-x, \end{aligned}$$

where $\langle \cdot ,\cdot \rangle$ is the standard inner product. We refer to [1] for further examples of retraction pairs for several manifolds of practical interest.

Given a retraction pair (*P*, *Q*), we can construct generalized quasi-interpolants $\mathbf {Q}^{(P,Q)}f(x)$ based on the first order condition (8), which defines a geometric average based on (*P*, *Q*) via

21 $$\begin{aligned} \sum _{i\in \mathcal {I}} \gamma _iQ\left( av_{P,Q}(\Gamma ,\mathcal {P}),p_i\right) = 0 \end{aligned}$$

The results in [15] show that this expression is locally well-defined. The construction above allows us to define a geometric quasi-interpolant based on an arbitrary retraction pair as follows.

#### Definition 3.12

Given a retraction pair (*P*, *Q*) and denoting $\Phi (x):=(\varphi _i(x))_{i\in \mathcal {I}}\subset \mathbb {R}$ and $f(\Xi ):=(f(\xi _i))_{i\in \mathcal {I}}\subset M$ we define the nonlinear moving least squares approximant

22 $$\begin{aligned} \mathbf {Q}^{(P,Q)}f(x):= av_{P,Q}(\Phi (x),\quad f(\Xi ))\in M. \end{aligned}$$

The following generalization of Theorem 3.5 holds true.

#### Theorem 3.13

Let $\Omega \subset \mathbb {R}^s$ be an open domain, $k>0$ a positive integer, $\Xi = (\xi _i)_{i\in \mathcal {I}}\subset \bar{\Omega }\subset \mathbb {R}^s$, $\Phi =(\varphi _i)_{i\in \mathcal {I}}\subset C_c(\bar{\Omega },\mathbb {R})$ and (*P*, *Q*) a retraction pair. Assume that $(\Xi ,\Phi )$ reproduces polynomials of degree smaller than *k*. Then there exists a constant $C>0$, depending only on *k* and *s*, such that for all $f\in C^k(\Omega ,M)$ and $x\in \Omega$ with $\Omega _x\subset \Omega$ and $\Theta _{k,\Omega _x}(f)<\infty$ we have

23 $$\begin{aligned}&\left| Q\left( \mathbf {Q}^{(P,Q)}f(x),f(x)\right) \right| _{g(\mathbf {Q}^{(P,Q)}f(x))} \nonumber \\&\quad \le C \Theta _{k,\Omega _x}(f) \sum _{i\in \mathcal {I}(x)}|\varphi _i(x)| \sup _{1\le r\le k}\sup _{y\in \Omega _x} \left\| \nabla _2^{r}Q\left( \mathbf {Q}^{(P,Q)}f(x),f(y)\right) \right\| h(x)^k. \end{aligned}$$

#### Proof

The proof is completely analogous to the proof of Theorem 3.5 with $\log$ replaced by *Q*. $\square$

## Numerical examples

In this section we describe the implementation and an application for our approximation operator $\mathbf {Q}^M$. In Sect. 4.1 we briefly explain for several manifolds how to compute the Riemannian average. In Sect. 4.2 we present two examples of approximations of manifold-valued functions. Finally in Sect. 4.3 we apply our approximation operator on parameter dependent linear time-invariant systems.

### Computation of Riemannian averages

In this section we briefly explain how the Riemannian average can be computed. For data points $\mathcal {P}=(p_i)_{i\in \mathcal {I}}\subset M$ with weights $(\gamma _i)_{i\in \mathcal {I}}\subset \mathbb {R}$ we use the iteration $\phi :M\rightarrow M$ defined by

24 $$\begin{aligned} \phi (p):=\exp \left( p,\sum _{i\in \mathcal {I}} \gamma _i \log (p,p_i)\right) . \end{aligned}$$

By (1.5.1)–(1.5.3) of [21] for data points close to each other this iteration converges linearly to the Riemannian average with convergence rate bounded by $\sup _{i,j\in \mathcal {I}} d^2(p_i,p_j)$ times a factor that depends only on the sectional curvature of *M*. If $\mathcal {P}=(p_i)_{i\in \mathcal {I}(x)}=(f(\xi _i))_{i\in \mathcal {I}(x)}$ and $f\in C^1(\Omega ,M)$ we have

$$\begin{aligned} d(p_i,p_j)=d(f(\xi _i),f(\xi _j))\le |f|_{C^1(\Omega ,M)}|\xi _i-\xi _j|\le |f|_{C^1(\Omega ,M)}2h(x). \end{aligned}$$

Hence, the convergence rate is bounded by $|f|^2_{C^1(\Omega ,M)}h(x)^2$ times a constant depending only on the sectional curvature of *M*. Therefore, our approximation operator $\mathbf {Q}^M$ satisfies (iv) from the introduction. To perform the iteration (24) we only need to know the exponential and logarithm map of the manifold. For the manifolds of practical interest there are explicit expressions for *log* and *exp* available. For the sphere we have for example

$$\begin{aligned} \exp (p,v)= & {} \cos (|v|)p+\frac{\sin (|v|)}{|v|}v \text { and}\\ \log (p,q)= & {} \frac{\arccos (\langle p,q\rangle ) }{\sqrt{1-\langle p,q\rangle ^2}}(q-\langle p,q\rangle p). \end{aligned}$$

With the usual metric on the space of SPD matrices (see e.g. [24]) the exponential and logarithm map are

$$\begin{aligned} \exp (P,Q)= & {} P^{1/2}{\text {Exp}}(P^{-1/2}QP^{-1/2})P^{1/2} \text { and}\\ \log (P,Q)= & {} P^{1/2}{\text {Log}}(P^{-1/2}QP^{-1/2})P^{1/2}. \end{aligned}$$

where ${\text {Exp}}$ denotes the matrix exponential and ${\text {Log}}$ the matrix logarithm. See [20, 25] for a survey of different methods for the computation of the Karcher mean of SPD matrices.

The exponential and logarithm map on the space of invertible matrices are

25 $$\begin{aligned} \exp (X,Y)={\text {Exp}}(Y)X \quad \text { and }\quad \log (X,Y)={\text {Log}}(YX^{-1}). \end{aligned}$$

An alternative way to compute the Karcher mean is to use Newton-like methods. See [22] for the computation of the Karcher mean on the sphere and the space of orthogonal matrices using Newton-like methods.

### Interpolation of a sphere-valued and a SPD-valued function

**Fig. 1:**
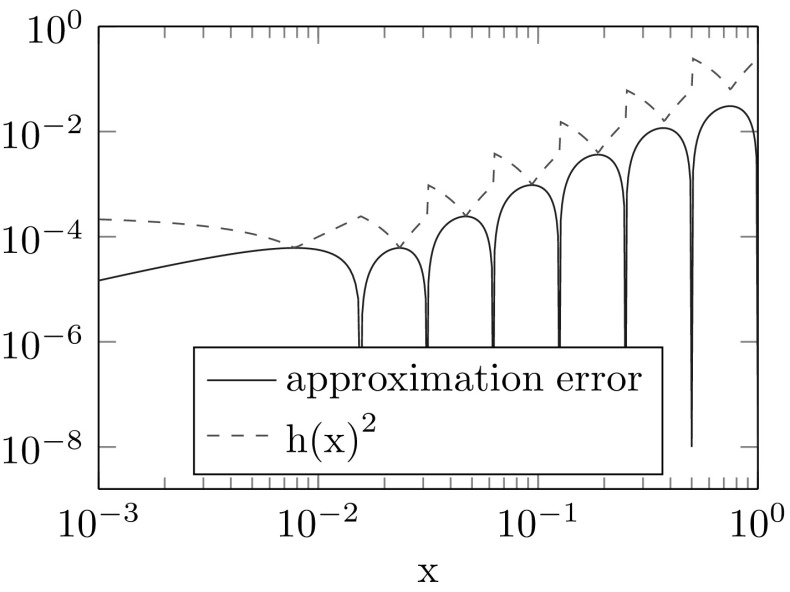
Approximation error for a sphere-valued function

**Fig. 2:**
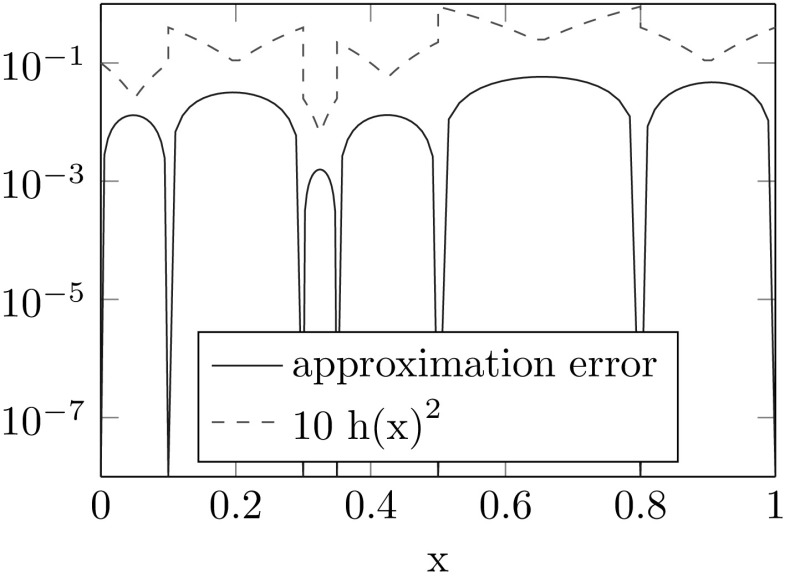
Approximation error for a SPD-valued function

In this section we present two examples for the interpolation of manifold-valued functions. For both interpolations we use the hat functions defined in Example 2.3. Then $Q^Mf$ is a piecewise geodesic function. Figures 1 and 2 illustrate that the approximation error can be bounded by a constant times the squared local meshwidth as stated in Theorem 3.9.

#### Example 4.1

We consider the function $f:[0,1] \rightarrow S^2$ defined by

$$\begin{aligned}{}[0,1] \ni x \mapsto \frac{ \begin{pmatrix} 1,x,x^2 \end{pmatrix} }{\left| \begin{pmatrix} 1,x,x^2 \end{pmatrix} \right| } \end{aligned}$$

and the nodes $\{0\} \cup \{2^{-j}|j\in \{0,1,\dots ,6 \}\}$.

#### Example 4.2

Let $\Omega =(0,1)$, $(\xi _1,\ldots ,\xi _7)=(0,0.1,0.3,0.35,0.5,0.8,1)$ and $f(x)=\cos (x\pi /2)A0+\sin (x\pi /2)A1$, where *A*0 and *A*1 are randomly chosen SPD matrices. To measure the error we used the geodesic distance $d(X,Y)=\Vert {\text {Log}}(X^{-1/2}YX^{-1/2})\Vert _{F}$, where *F* denotes the Frobenius norm.

### Approximation of reduced order models (ROMs)

In [6], Amsallem and Farhat present a fast method for solving parameter dependent linear time-invariant (LTI) systems. They assume that the solution is known for a few sets of parameters and interpolate the matrices of a reduced order models to get the matrix of a reduced order model for a new set of parameters. Some of these matrices live in a nonlinear space. They choose the ‘push-interpolate-pull’ technique to interpolate the data. We present an adaptation of this method, based upon the theory in this paper, for approximating reduced order models (ROMs). As we will see in some cases our adaptation yields reliable result whereas the original ROM approximation method fails.

We start by introducing linear time invariant systems. Then we present the ROM approximation method and our adaptation.

#### Linear time-invariant systems

In a parameter dependent LTI system as in [6] we assume that for each $x \in \Omega$ there exists a unique solution $z_x :[0,T] \rightarrow \mathbb {R}^q$ of

26 $$\begin{aligned} \frac{dw_x}{dt}(t)= & {} A(x) w_x(t)+B(x) u(t),\end{aligned}$$

27 $$\begin{aligned} z_x(t)= & {} C(x)w_x(t)+D(x)u(t), \end{aligned}$$

where $A:\Omega \rightarrow GL(n)$, with *GL*(*n*) the set of invertible matrices of size $n \times n$, $B:\Omega \rightarrow \mathbb {R}^{n \times p},\ C :\Omega \rightarrow \mathbb {R}^{q \times n},\ D:\Omega \rightarrow \mathbb {R}^{q \times p}$ and $u:[0,T] \rightarrow \mathbb {R}^p$. Typically $z_x$ is an output functional of a dynamical system with control function *u* and $n \gg p,q$. Furthermore we assume that *A*, *B*, *C* and *D* are continuous. For $k<n$ we define the compact Stiefel manifold by

$$\begin{aligned} St(n,k):=\{U \in \mathbb {R}^{n \times k}\ |\ U^TU=I_k\}, \end{aligned}$$

where $I_k$ denotes the $k\times k$ identity matrix. Let $U,V:\Omega \rightarrow St(n,k)$ define test and trial bases, respectively. The state vector $w_x(t)$ will be approximated as a linear combination of column vectors of *V*(*x*), i.e. $w_x(t) \approx V(x)\bar{w}_x(t)$ where $\bar{w}_x$ will be defined by substituting $w_x$ by $V(x)\bar{w}_x$ and multiplying Eq. (26 27) from the left by $U(x)^T$. Hence we get the system of equations

28 $$\begin{aligned} U^TV(x)\frac{d\bar{w}_x}{dt}(t)= & {} U^TAV(x)\bar{w}_x(t)+U^TB(x) u(t), \end{aligned}$$

29 $$\begin{aligned} \bar{z}_x(t)= & {} CV(x)\bar{w}_x(t)+D(x)u(t), \end{aligned}$$

where all operations on matrix-valued functions are defined pointwise. Multiplying Eq. (28 29) from the left by $(U^TV(x))^{-1}$ yields the new LTI system

30 $$\begin{aligned} \frac{d\bar{w}_x}{dt}(t)= & {} \mathcal {A}(x) \bar{w}_x(t)+\mathcal {B}(x) u(t),\end{aligned}$$

31 $$\begin{aligned} \bar{z}_x(t)= & {} \mathcal {C}(x)\bar{w}_x(t)+\mathcal {D}(x)u(t), \end{aligned}$$

where $\mathcal {A}:\Omega \rightarrow \mathbb {R}^{k \times k}$, $\mathcal {B}:\Omega \rightarrow \mathbb {R}^{k \times p}$, $\mathcal {C}:\Omega \rightarrow \mathbb {R}^{q \times k}$ and $\mathcal {D}:\Omega \rightarrow \mathbb {R}^{q \times p}$ are

32 $$\begin{aligned} \mathcal {A}:= & {} (U^TV)^{-1}U^TAV,\end{aligned}$$

33 $$\begin{aligned} \mathcal {B}:= & {} (U^TV)^{-1}U^T, \end{aligned}$$

34 $$\begin{aligned} \mathcal {C}:= & {} CV\text { and}\end{aligned}$$

35 $$\begin{aligned} \mathcal {D}:= & {} D. \end{aligned}$$

The aim is that $\bar{z}_x$ has the same properties and features as $z_x$. Furthermore *k* should be much smaller than *n* so that $\bar{z}_x(t)$ can be computed (with the help of the matrices $\mathcal {A}$, $\mathcal {B}$, $\mathcal {C}$ and *D*) significantly faster than $z_x(t)$.

Let $O(k):=\{Q \in \mathbb {R}^{k \times k}|QQ^T=I_k \}$ be the space of orthogonal matrices. Note that a coordinate transformation $\bar{U}(x)=UQ(x)$ where $Q(x) \in O(k)$ is an orthogonal matrix does not change the matrices $\mathcal {A},\dots ,\mathcal {D}$ nor the solution $\bar{z}_x$. Similarly a coordinate transformation $\bar{V}(x)=VQ(x)$ where $Q(x) \in O(k)$ is an orthogonal matrix transforms the solution $\bar{z}_x$ isometrically. We therefore introduce the equivalence relation

36 $$\begin{aligned} U \sim \bar{U} :\Leftrightarrow \exists Q:\Omega \rightarrow O(k) \text { s.t. } \bar{U}=UQ. \end{aligned}$$

The corresponding space *St*(*n*, *k*) / *O*(*k*) is also known as the Grassmannian.

Given a parameter dependent LTI-system and assume that for each choice of the parameter $x\in \Omega$ there exists a ROM with matrices $U(x),V(x),\mathcal {A}(x),\mathcal {B}(x),\mathcal {C}(x)$. Furthermore assume that the maps $x\mapsto \Pi (U(x))$ and $x\mapsto \Pi (V(x))$ where $\Pi :St(n,k) \rightarrow St(n,k) / O(k)$ denotes the natural projection, are continuous. The maps *U*, *V*, $\mathcal {A}$, $\mathcal {B}$ and $\mathcal {C}$ on the other hand do not have to be continuous. The problem of interpolating reduced-order models (ROMs) is: given the reduced order models $(\mathcal {A}(\xi _i),\mathcal {B}(\xi _i),\mathcal {C}(\xi _i))$ for several parameters $\xi _i,\; i \in \mathcal {I}$ and $x\in \Omega$ find an approximation for the reduced order model $(\mathcal {A}(x),\mathcal {B}(x),\mathcal {C}(x))$.

As we are aiming for a fast method the running time should be independent of *n*. In addition to the matrices $(\mathcal {A}(\xi _i),\mathcal {B}(\xi _i),\mathcal {C}(\xi _i))$ we can also use precomputed matrices of size $\ll n$.

#### The ROM approximation method

We sketch the method proposed by Amsallem and Farhat in [6]. The algorithm is divided into two steps. In the first step we construct a continuous function $V^c:\Omega \rightarrow St(n,k)$ with $V^c\sim V$. To this end we choose $V_0\in \mathbb {R}^{n \times k}$ such that $V(x)^TV_0\in \mathbb {R}^{k\times k}$ is invertible for all $x\in \Omega$. In [6] the matrix $V_0$ is chosen by $V_0:=V(\xi _{i})$ for some $i \in \mathcal {I}$. Then we define *f* by

37 $$\begin{aligned} f(V)(x):=V(x)P(x):=V(x)\mathcal {P}_{O(k)}(V(x)^TV_0), \end{aligned}$$

for all $V:\Omega \rightarrow St(n,k)$ where $\mathcal {P}_{O(k)}$ denotes the closest point projection onto *O*(*k*) defined below.

##### Definition 4.3

We define the closest point projection $\mathcal {P}_{O(k)}:GL(k) \rightarrow O(k)$ by

$$\begin{aligned} \mathcal {P}_{O(k)}(X):={{\mathrm{arg\,min}}}_{Y \in O(k)} \Vert X-Y\Vert _F. \end{aligned}$$

To compute the closest point projection onto *O*(*k*) in a stable way, one can use the iteration (see e.g. [18])

$$\begin{aligned} \phi (X)=\frac{X+X^{-T}}{2}. \end{aligned}$$

The next lemma shows that *f* can be regarded as a map from $\Omega$ into *St*(*n*, *k*) / *O*(*k*).

##### Lemma 4.4

If $(\tilde{V},V)$ we have

$$\begin{aligned} f(\tilde{V})=f(V). \end{aligned}$$

##### Proof

By the definition of the equivalence relation (36) there exists $Q:\Omega \rightarrow O(k)$ such that $\tilde{V}=VQ$. Note that it is enough to prove that

$$\begin{aligned} Q(x)\mathcal {P}_{O(k)}(\tilde{V}(x)^TV_0)=\mathcal {P}_{O(k)}(V(x)^TV_0) \end{aligned}$$

for all $x\in \Omega$. By left invariance of the closest point projection with respect to orthogonal matrices we have

$$\begin{aligned} Q(x)\mathcal {P}_{O(k)}(\tilde{V}(x)^TV_0)= & {} Q(x)\mathcal {P}_{O(k)}(Q(x)^TV(x)^TV_0)\\= & {} QQ^T(x)\mathcal {P}_{O(k)}(V(x)^TV_0)\\= & {} \mathcal {P}_{O(k)}(V(x)^TU_0). \end{aligned}$$

$$\square $$

Next we prove that *f*(*V*) has the same smoothness as $\Pi (V)$.

##### Proposition 4.5

Assume that $\Pi (V):\Omega \rightarrow St(n,k)/O(k)$ is *k* times differentiable. Then the map $f(V):\Omega \rightarrow St(n,k)$ is *k*-times differentiable as well.

##### Proof

By the assumption there exists a *k* times differentiable function $\bar{V}$ with $\bar{V}\sim V$. By Lemma 4.4 we have $f(V)=f(\bar{V})$. By the smoothness of the closest point projection we have that $f(\bar{V})$ is *k* times differentiable. $\square$

Replacing *V* by $V^c=f(V)$ in (32 33 34 35) we can define continuous matrix-valued functions $\mathcal {A}^c,\mathcal {B}^c,\mathcal {C}^c$ for the reduced order models by

$$\begin{aligned} \begin{array}{lllll} \mathcal {A}^c &{}:=&{} (U^TV^c)^{-1}U^TAV^c&{}=&{} P^T\mathcal {A}P,\\ \mathcal {B}^c&{}:=&{} (U^TV^c)^{-1}U^T B&{}=&{} P^T \mathcal {B}\quad \text { and}\\ \mathcal {C}^c&{}:=&{} CV^c&{}=&{} \mathcal {C}P, \end{array} \end{aligned}$$

where $P(x)=\mathcal {P}_{O(k)}(V(x)^TV_0)$ for all $x\in \Omega$. In step 2 the data $\mathcal {A}^c(x)$ is approximated with respect to the space *GL*(*k*) [see (25) for the logarithm and exponential map on *GL*(*k*)].

The ROM approximation algorithm interpolates the data $\mathcal {A}^c(\xi _{i})$ using the ‘push-interpolate-pull’ technique, i.e. it chooses an $i_0 \in \mathcal {I}$ and maps the data $\mathcal {A}^c(\xi _{i})$ by the logarithm map $\log$ with base point $\mathcal {A}^c(\xi _{i_0})$ to the tangent space at $\mathcal {A}^c(\xi _{i_0})$. Then the new data is approximated with a method for linear spaces and finally mapped back to the manifold by the exponential map $\exp$.

The data $\mathcal {B}^c(x)$ and $\mathcal {C}^c(x)$ is approximated with a method for linear spaces.

We present an adaptation of the ROM approximation algorithm by using the approximation operator $\mathbf {Q}^M$ defined in 3.2 to interpolate the data $\mathcal {A}(x)$ in *GL*(*k*).
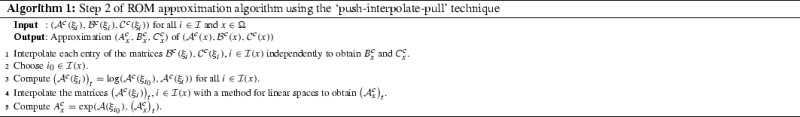
 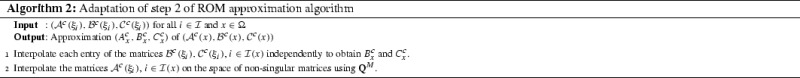


#### Numerical experiments

In Section 5.1 of [6] a simple academic example where the ROM approximation method yields good results is shown. In the next example we can only get a reasonable approximation if we use our adaptation.

##### Example 4.6

**Fig. 3:**
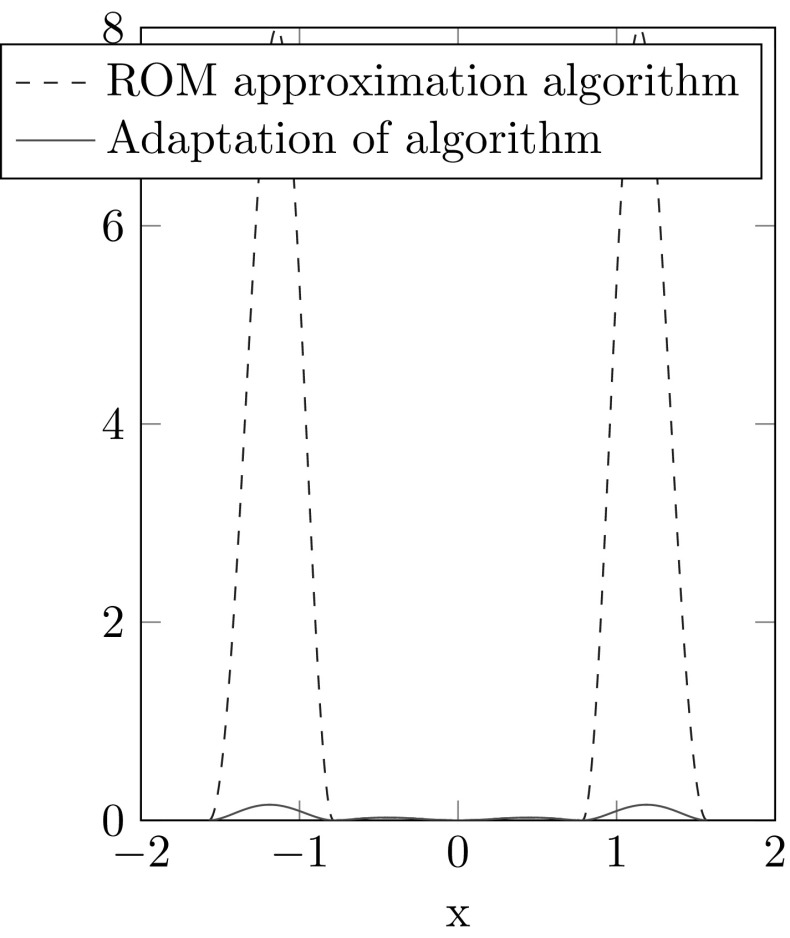
Error plot of ROM approximation algorithm and its adapted version

**Fig. 4:**
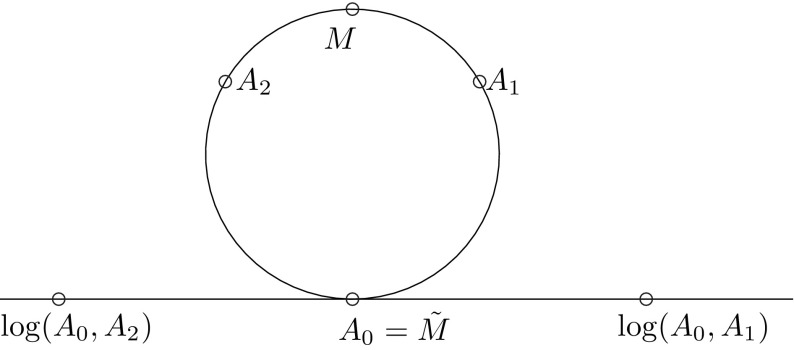
Illustration for the explanation of the large error in Fig. 3

In this example we consider an interpolation of LTI-systems without a reduction. Hence we can set $(\mathcal {A},\mathcal {B},\mathcal {C})=(A,B,C)$ and omit step 1 of the ROM Approximation Algorithm. Let

$$\begin{aligned} A(x):= \begin{pmatrix} \cos (g(x)) \qquad \sin (g(x) \\ -\sin (g(x) \qquad \cos (g(x)) \end{pmatrix}, \end{aligned}$$

where $g(x)=4\sin (\pi x)$ and $\xi _i=\frac{i}{4}$ for $i \in \{-2,-1,0,1,2\}$. We choose $i_0=0$ and the hat functions $\varphi _i(x)$ from Example 2.3 as basis functions. The error for *A* measured in the Frobenius norm for the ROM-Approximation and its adapted algorithm are illustrated in Fig. 3. An illustration why the ROM approximation method has a large error is shown in Fig. 4. As the matrices *A*(*x*) are two dimensional rotation matrices we can see them as points on a circle. The Riemannian average of points $A_1$ and $A_2$ with weights $\lambda _1=\lambda _2=0.5$ is *M*. However if we transform the points by the logarithm to the tangent space at $A_0$, interpolate on this tangent space and transform back by the exponential map we get a different point $\tilde{M}\ne M$.

##### Example 4.7

We consider the values $n=3,\ p=q=1$ and the matrices $A(x):=A_0+xA_1$, $B(x):=B$ and $C(x):=C$ for all $x\in [-1,1]$ where

$$\begin{aligned} A_0:= \begin{pmatrix} 6 \quad 4 \quad 2\\ 8 \quad 4 \quad 2\\ 12 \quad 4 \quad 20 \end{pmatrix},\; A_1:= \begin{pmatrix} 1 \quad 2 \quad 3\\ 4 \quad 5 \quad 6\\ 3 \quad 2 \quad 1 \end{pmatrix},\;B:= \begin{pmatrix} 1\\ 0 \\ 0 \end{pmatrix} \text { and } C:= \begin{pmatrix} 1 \quad 2 \quad 3 \end{pmatrix}. \end{aligned}$$

**Fig. 5:**
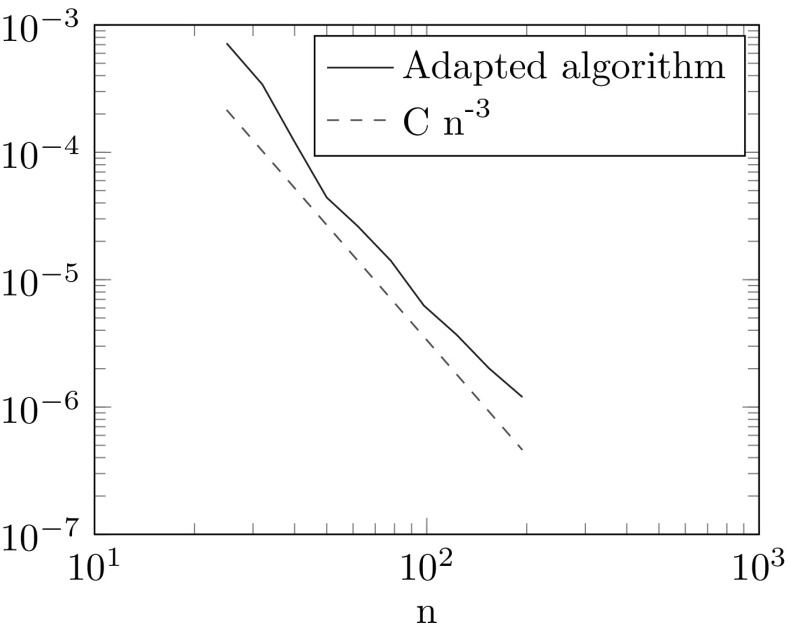
Convergence plot for error made in Algorithm 2

We set *U*(*x*) and *V*(*x*) equal to the first 2 columns of the orthogonal matrices of the singular value decomposition of *A*(*x*). We choose the data sites from Example 2.8 with $k=2$. The $L^\infty$-norm of the error made in Step 2 of Algorithm 2 is shown in Fig. 5. As we can see the convergence rate is as predicted by Theorem 3.9.
